# Molecular pathogen screening of louse flies (Diptera: Hippoboscidae) from domestic and wild ruminants in Austria

**DOI:** 10.1186/s13071-023-05810-4

**Published:** 2023-06-02

**Authors:** Miguel Peña-Espinoza, Daniel Em, Bita Shahi-Barogh, Dominik Berer, Georg G. Duscher, Lara van der Vloedt, Walter Glawischnig, Steffen Rehbein, Josef Harl, Maria S. Unterköfler, Hans-Peter Fuehrer

**Affiliations:** 1 Institute of Parasitology, Department of Pathobiology, University of Veterinary Medicine Vienna, Vienna, Austria; 2 Austrian Agency for Health and Food Safety (AGES), Research Services, Vienna, Austria; 3 Austrian Agency for Health and Food Safety (AGES), Institute for Veterinary Disease Control, Innsbruck, Austria; 4 Boehringer Ingelheim Vetmedica GmbH, Kathrinenhof Research Center, Rohrdorf, Germany; 5 Institute of Pathology, Department of Pathobiology, University of Veterinary Medicine Vienna, Vienna, Austria

**Keywords:** *Bartonella*, *Hippobosca equina*, Hippoboscidae, *Lipoptena cervi*, *Melophagus ovinus*, Vector-borne pathogens, Barcoding, Louse flies, Keds, Ruminants

## Abstract

**Background:**

Hippoboscid flies (Diptera: Hippoboscidae), also known as louse flies or keds, are obligate blood-sucking ectoparasites of animals, and accidentally of humans. The potential role of hippoboscids as vectors of human and veterinary pathogens is being increasingly investigated, but the presence and distribution of infectious agents in louse flies is still unknown in parts of Europe. Here, we report the use of molecular genetics to detect and characterize vector-borne pathogens in hippoboscid flies infesting domestic and wild animals in Austria.

**Methods:**

Louse flies were collected from naturally infested cattle (*n* = 25), sheep (*n* = 3), and red deer (*n* = 12) across Austria between 2015 and 2019. Individual insects were morphologically identified to species level and subjected to DNA extraction for molecular pathogen screening and barcoding. Genomic DNA from each louse fly was screened for *Borrelia* spp., *Bartonella* spp., Trypanosomatida, Anaplasmataceae, Filarioidea and Piroplasmida. Obtained sequences of Trypanosomatida and *Bartonella* spp. were further characterized by phylogenetic and haplotype networking analyses.

**Results:**

A total of 282 hippoboscid flies corresponding to three species were identified: *Hippobosca equina* (*n* = 62) collected from cattle, *Melophagus ovinus* (*n* = 100) from sheep and *Lipoptena cervi* (*n* = 120) from red deer (*Cervus elaphus*). Molecular screening revealed pathogen DNA in 54.3% of hippoboscids, including infections with single (63.39%), two (30.71%) and up to three (5.90%) distinct pathogens in the same individual. *Bartonella* DNA was detected in 36.9% of the louse flies. *Lipoptena cervi* were infected with 10 distinct and previously unreported *Bartonella* sp. haplotypes, some closely associated with strains of zoonotic potential. DNA of trypanosomatids was identified in 34% of hippoboscids, including the first description of *Trypanosoma* sp. in *H. equina*. Anaplasmataceae DNA (*Wolbachia* spp.) was detected only in *M. ovinus* (16%), while < 1% of the louse flies were positive for *Borrelia* spp. and Filarioidea. All hippoboscids were negative for Piroplasmida.

**Conclusions:**

Molecular genetic screening confirmed the presence of several pathogens in hippoboscids infesting domestic and wild ruminants in Austria, including novel pathogen haplotypes of zoonotic potential (e.g. *Bartonella* spp.) and the first report of *Trypanosoma* sp. in *H. equina*, suggesting a potential role of this louse fly as vector of animal trypanosomatids. Experimental transmission studies and expanded monitoring of hippoboscid flies and hippoboscid-associated pathogens are warranted to clarify the competence of these ectoparasites as vectors of infectious agents in a One-Health context.

**Graphical Abstract:**

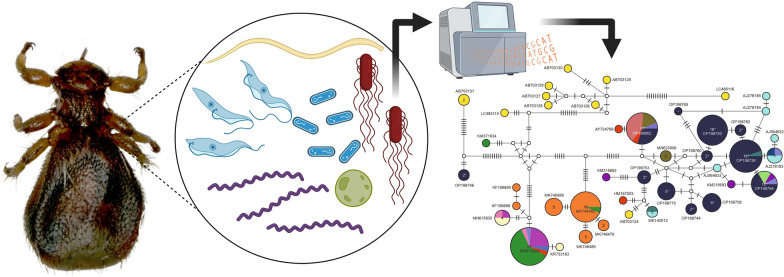

**Supplementary Information:**

The online version contains supplementary material available at 10.1186/s13071-023-05810-4.

## Background

Hippoboscid flies (Diptera: Hippoboscidae), also known as louse flies or keds, are obligatory blood-sucking ectoparasites infesting mammals and birds worldwide [1]. To date, most research on hippoboscids has focused on understanding their biology, evolution, host specificity and impact of their hematophagous and biting behavior on animals and humans [2–8]. Various louse fly species of the genera *Melophagus* spp., *Lipoptena* spp. and *Hippobosca* spp. have been described to commonly infest domestic and wild ungulates in Europe [9–11], and occasionally also attack humans and pets [12–15]. Indeed, it appears that hippoboscid flies may have been attacking humans for millennia, as suggested by the identification of the common deer ked *Lipoptena cervi* on the late neolithic human mummy “Ötzi” in the Ötztal Alps [16]. Considering their blood-feeding nature, widespread distribution and the broad host spectrum of some species, hippoboscid flies may also act as potential vectors of infectious diseases within animal populations, and between animals and humans [17].

Hippoboscid flies have been investigated for their role as vectors of animal pathogens for over a century [18, 19], with molecular studies in the last 2 decades confirming several hippoboscid-associated pathogens of medical and veterinary importance in different louse fly species [17]. A wide range of vector-borne bacteria and protozoa have been identified in hippoboscid flies collected from domestic and wild ruminants in some European countries, including *Anaplasma* spp., *Babesia* spp.*, Bartonella* spp., *Borrelia* spp., *Mycoplasma* spp.*, Rickettsia* spp., *Theileria* spp. and *Trypanosoma* spp. [20–31]. Despite these research efforts, there are still major knowledge gaps regarding the presence and monitoring of emerging vector-borne diseases in hippoboscid flies in Europe, including Austria. Moreover, in view of the widespread distribution of free-ranging wild ruminants that can act as reservoirs of infectious agents in Austria [32, 33] and the increasing human presence in areas populated by wild animals due to working or leisure activities, the vector role of hippoboscids warrants further elucidation.

The aim of the present study was to detect the presence of vector-borne pathogens in hippoboscid flies infesting domestic and wild ruminants in Austria using molecular techniques. In addition, DNA barcoding of the hippoboscid flies was performed to confirm and characterize their identity.

## Materials and methods

### Study areas and collection of hippoboscid flies

**Fig. 1:**
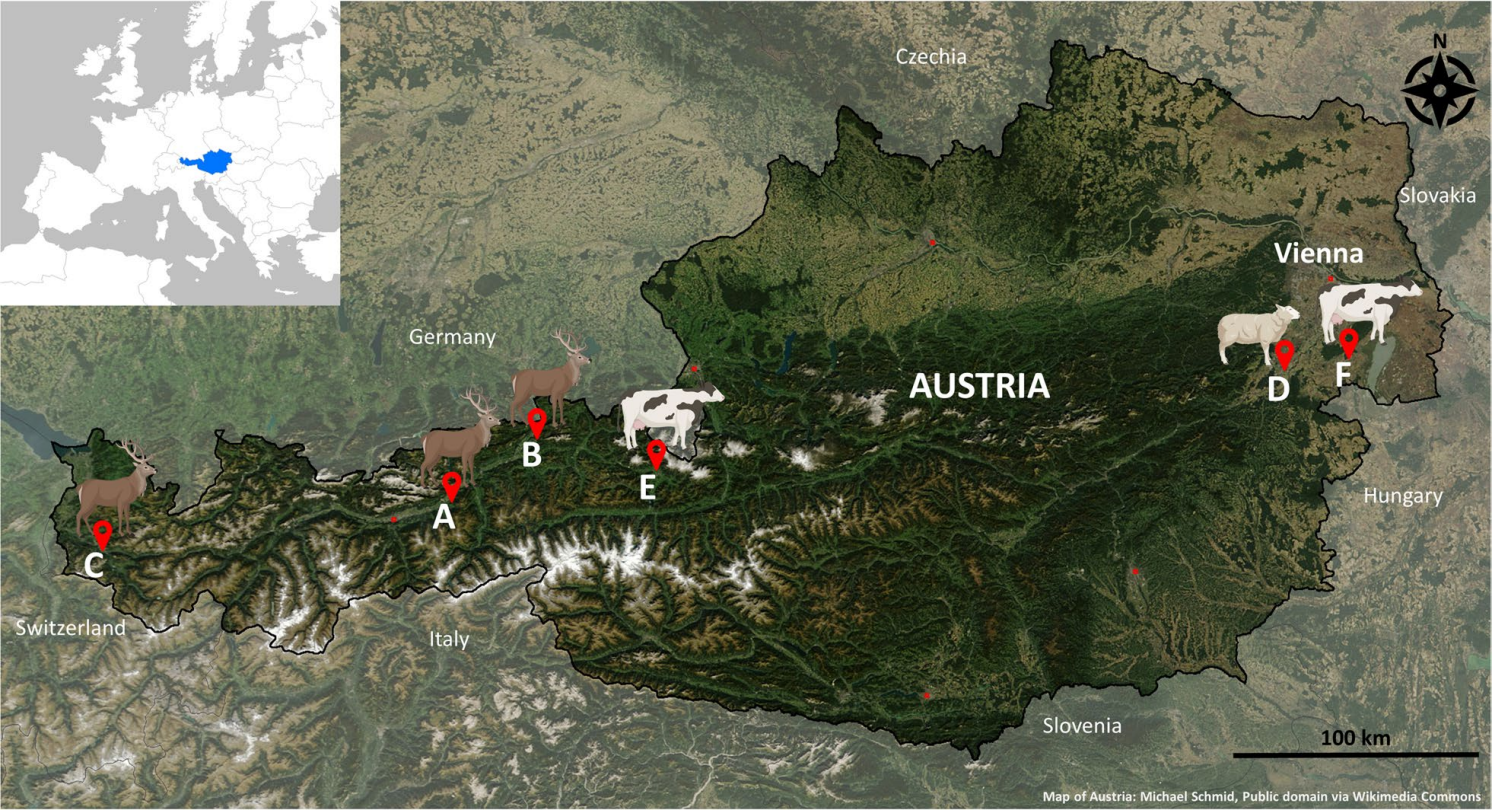
Origin of hippoboscid flies from Austria. Collection sites of hippoboscid flies from domestic and wild ruminants in Schwaz (**A**), Kufstein (**B**), Bludenz (**C**), Leobersdorf (**D**), Saalfelden (**E**) and Eisenstadt (**F**), in Austria. See the main text for further information

Hippoboscid flies were collected from red deer (*Cervus elaphus;* *n* = 12), sheep (*Ovis aries*; *n* = 3) and cattle (*Bos taurus*; *n* = 25) in various locations in Austria, between 2015 and 2019 (Fig. 1). Hippoboscids infesting red deer were sampled in November/December of 2016 and 2017 from hunted animals at three sites: Schwaz (Fig. 1A) and Kufstein (Fig. 1B) in the Federal State of Tyrol and Bludenz (Fig. 1C) in the Federal State of Vorarlberg. Hunted red deer from these areas are routinely examined as part of the tuberculosis surveillance in wildlife by the Austrian Agency for Health and Food Safety (AGES). Hippoboscids were collected from the head skin of 12 recently hunted red deer submitted to the AGES Laboratory, with an estimated presence of deer keds in 20–30% of all hunted red deer investigated in the surveillance program (W. Glawischnig, personal communication). A total of 120 louse flies were sampled from the examined animals. Louse flies from sheep were obtained in March 2018 directly at a farm in Leobersdorf, Federal State of Lower Austria (Fig. 1D). At sampling, the farm had a herd of 30 adult sheep, with an observed presence of keds in 100% of the animals. A total of 100 sheep keds were collected directly from 3 adult sheep during shearing. Hippoboscids from cattle were collected from grazing animals in July/August of 2016 and 2017 in the Saalfelden area, Federal State of Salzburg (Fig. 1E), in the course of a 2-year epidemiological study involving inspection of the animals at regular intervals during the grazing season [34]. Per occasion, 31 to 57 cattle were visually examined, and louse flies were observed in up to 33% of the cattle (peak of infestation in August). A total of 61 hippoboscid individuals were collected from 24 cattle from Saalfelden between 2016 and 2017 (with some louse flies being collected from the same animal) after visual identification of the insects on the cattle’s haircoat. An additional louse fly from cattle was collected in Eisenstadt in July 2019 (Federal Sate of Burgenland, Fig. 1F). At all sampling sites, hippoboscids were separated from hair/wool manually or using fine forceps and stored immediately either dry or in ethanol in individual Eppendorf tubes. All hippoboscids were identified to species level using a stereomicroscope (Nikon SMZ1270, Tokyo, Japan) and morphological keys [35, 36], followed by DNA extraction.

### DNA extraction

The individual hippoboscids were subjected to total DNA extraction for molecular pathogen screening and insects’ barcoding. Individual hippoboscids were mixed with 180 μl buffer ATL (DNeasy Blood & Tissue Kits, Qiagen) in 1.5-ml Eppendorf tubes, and two 1.4-mm ceramic beads (Qiagen, Hilden, Germany) were added per tube, followed by mechanical homogenization in a TissueLyser II (Qiagen, Hilden, Germany) at room temperature for 6 min. Then, 20 μl proteinase K was added, and the tubes were vortexed and incubated at 56 °C overnight. After incubation, total DNA was extracted from insect material using the QIAGEN DNeasy Blood & Tissue Kit (QIAGEN, Hilden, Germany), following the manufacturer’s instructions.

### Barcoding of hippoboscid flies

To confirm the species identity and to explore the genetic diversity of the collected hippoboscids, selected specimens were subjected to DNA barcoding analysis. Total DNA extracted from 21 hippoboscids was used to amplify a region within the insects’ mitochondrial *COI* by conventional PCR [37] as described in Table 1. The PCR products were sequenced at LGC Genomics GmbH (Berlin, Germany). The resulting *COI* sequences were used for the taxonomical characterization of the hippoboscid species by comparison with available sequences on the GenBank nucleotide database for organism identification using BLAST (https://blast.ncbi.nlm.nih.gov/Blast.cgi) and BOLD (www.boldsystems.org, accessed on 01 June 2022).

**Table 1:** Primers and PCR cycle conditions used for the molecular characterization of pathogens and hippoboscid flies (Diptera: Hippoboscidae) collected from domestic and wild ruminants in Austria

Organism	Target gene	Primers	Sequence (5'–3')	Length (bp)	PCR cycle conditions	References
Anaplasmataceae	*16S* rRNA	EHR16SD_for	GGT ACC YAC AGA AGA AGT CC	345	95 °C/2 min; 35 cycles: 94 °C/1 min, 54 °C/30 s, 72 °C/30 s; 72 °C/5 min	[40]
		EHR16SR_rev	TAG CAC TCA TCG TTT ACA GC			
*Borrelia*	*16S* rRNA	Borr_allg_for	ACG CTG GCA GTG CGT CTT AA	674	94 °C/2 min; 6 cycles: 94 °C/1 min, 45 °C/1.5 min, 72 °C/75 s; 36 cycles: 94 °C/1 min, 51 °C/1.5 min, 72 °C/75 s); 72 °C/5 min	[39]
		Borr_allg_rev	CTG ATA TCA ACA GAT TCC ACC C			
*Bartonella*	*gltA*	BhCs.781p	GGG GAC CAG CTC ATG GTG G	379	94 °C/5 min; 40 cycles: 94 °C/1 min, 54 °C/1 min, 72 °C/1 min; 72 °C/10 min	[38]
		BhCs.1137n	AAT GCA AAA AGA ACA GTA AAC A			
Filarioidea	*COI*	COlint-F	TGA TTG GTG GTT TTG GTA A	668	94 °C/2 min; 8 cycles: 94 °C/45 s, 51 °C/45 s, 72 °C/1.5 min; 25 cycles: 94 °C/45 s, 45 °C/45 s, 72 °C/1.5 min; 72 °C/7 min	[42]
		COlint-R	ATA AGT ACG AGT ATC AAT ATC			
Trypanosomatida	*18S* rRNA	Tryp_18S_F1 (Nest 1)	GTGGACTGCCATGGCGTTGA	960	94 °C/5 min; 35 cycles: 94 °C/1 min, 56 °C/1 min, 72 °C/1 min; 72 °C/5 min (Nest 1)	This study
		Tryp_18S_R1 (Nest 1)	CAGCTTGGATCTCGTCCGTTGA			
		Tryp_18S_F2 (Nest 2)	CGATGAGGCAGCGAAAAGAAATAGAG		94 °C/5 min; 25 cycles: 94 °C/1 min, 56 °C/1 min, 72 °C/1 min; 72 °C/5 min (Nest 2)	
		Tryp_18S_R2 (Nest 2)	GACTGTAACCTCAAAGCTTTCGCG			
Piroplasmida	*18S* rRNA	BTH-1F (Nest 1)	CCT GAG AAA CGG CTA CCA CAT CT	561	94 °C/2 min; 40 cycles: 95 °C/30 s, 68 °C/1 min, 72 °C/1 min; 72 °C/10 min (Nest 1)	[41]
		BTH-1R (Nest 1)	TTG CGA CCA TAC TCC CCC CA			
		G-2_for (Nest 2)	GTC TTG TAA TTG GAA TGA TGG		94 °C/2 min; 40 cycles: 95 °C/30 s, 60 °C/1 min, 72 °C/1 min; 72 °C/10 min (Nest 2)	
		G-2_rev (Nest 2)	CCA AAG ACT TTG ATT TCT CTC			
Lepidoptera	*COI*	LepF1	ATT CAA CCA ATC ATA AA	648	94 °C/2 min; 6 cycles: 94 °C/1 min, 45 °C/1.5 min, 72 °C/75 s; 36 cycles: 94 °C/1 min, 51 °C/1.5 min, 72 °C/75 s); 72 °C/5 min	[37]
		LepR1	TAA ACT TCT GGA TGT CAA AAA			

### Molecular pathogen screening by conventional and nested PCR

The obtained DNA from each hippoboscid fly was screened for the presence of several vector-borne pathogens by targeting selected genes using primers and PCR protocols summarized in Table 1. The hippoboscids were screened by conventional PCR for bacteria of the family Anaplasmataceae (*16S* ribosomal RNA), the genus *Borrelia* (*16S* ribosomal RNA) and the genus *Bartonella* (*citrate synthase* gene—*glt*A), as well as for nematodes of the superfamily Filarioidea (mitochondrial *cytochrome c oxidase subunit I* gene—*COI*). In addition, the DNA was screened for parasites of the orders Trypanosomatida (*18S* ribosomal RNA) and Piroplasmida (*18S* ribosomal RNA) using nested PCRs. The PCR methodologies were based on previously published protocols [38–42], except for the nested PCR protocol for Trypanosomatida, which was designed for the present study. The latter primers were designed based on all *18S* sequences of Trypanosomatida available on GenBank and allow the amplification of all strains. All PCR reactions were performed in an Eppendorf Mastercycler Pro (Eppendorf AG, Hamburg, Germany). The PCR products were stored at 15 °C until confirmation of the amplified regions of interest by electrophoresis in 2% agarose gels stained with Midori Green Advanced dye (Biozym Scientific, Germany). PCR products positive for the investigated pathogens were sequenced at LGC Genomics GmbH (Berlin, Germany) using amplification primers. The sequences were assembled with BioEdit [43] and compared to sequences available on NCBI GenBank (National Center for Biotechnology Information; https://blast.ncbi.nlm.nih.gov/Blast.cgi) using multiple BLAST searches.

### Phylogenetic and haplotype networking analyses of Trypanosomatida and *Bartonella* spp.

Selected sequences of Trypanosomatida and *Bartonella* spp. isolated from the investigated hippoboscids were subjected to phylogenetic analyses as previously described [44], with modifications. The sequences were aligned and cut to primer binding regions, and the electropherograms were manually checked for double peaks. Double peaks were identified in 19/27 *Bartonella* spp. sequences, which suggested a co-infection with two distinct strains of *Bartonella* sp. in the same insects. In those cases, the two strains were unphased to obtain single sequences and uploaded as individual sequences to GenBank. Each strain was separately uploaded to GenBank (acc. no. ON637624—ON637640 for Trypanosomatida; OP198738—OP198806 for *Bartonella* spp.) and used for phylogenetic analysis.

To provide an overview of the genetic diversity of detected (and related) Trypanosomatida and *Bartonella* spp. strains, maximum likelihood (ML) and Bayesian inference (BI) trees were calculated for each of the two groups based on alignments including 409 sequences (991 nucleotide positions) for *Trypanosoma* spp. and 582 sequences (338 nucleotide positions) for *Bartonella* spp. Gaps in the alignments were removed using TrimAl v.1.3 [45], and the sequences were collapsed to haplotypes using DAMBE v.7.0.5.1 [46], leaving 167 haplotypes (701 nucleotide positions) for *Trypanosoma* spp. and 261 haplotypes for *Bartonella* spp. As outgroup of the *Trypanosoma* spp. tree, a sequence of *Belchomonas wendygibsoni* (KF054126) was used. No suitable sequence was available as outgroup for *Bartonella* spp., and this tree was instead mid-point rooted. The ML bootstrap consensus trees (1000 replicates) were calculated using the W-IQ-TREE web server [47] and applying the models TIM3e + I + G4 for *Trypanosoma* spp. and JC for *Bartonella* spp., which were suggested as best fit for the data set in the model test according to the corrected Akaike information criterion. The BI trees were calculated using MrBayes v.3.2.7 [48] applying the next complex model GTR + G + I for *Trypanosoma* spp. and JC for *Bartonella* spp. The analyses were run for 10,000,000 generations (number of chains: 4), sampling every thousandth tree. The first 25% of trees were discarded as burn-in, and a 50% majority-rule consensus tree was calculated based on the remaining 7500 trees. The sequences for the DNA haplotype network analyses were selected based on well-supported clades in the phylogenetic trees (see Additional file 1: Figs. S1 and S2). Median-joining haplotype networks were calculated with Network 10.2.0.0 (Fluxus Technology Ltd., Suffolk, UK), applying the default settings. The networks were graphically prepared and provided with information on the countries and hosts in Network Publisher v.2.1.2.5 (Fluxus Technology Ltd., Suffolk, UK) and finalized with CorelDRAW 2021 (Corel, Ottawa, Canada).

### Data analysis

Data processing and descriptive statistics were performed in Microsoft Excel and GraphPad Prism 7. Statistical analyses were implemented in R version 4.0.3 [49]. Differences in total infection rate (all pathogen groups combined) and in infection prevalence of each pathogen group among the three hippoboscid species were evaluated by the test of equal or given proportions (*prop.test*) and pairwise comparison for proportions with Holm-Bonferroni method (*pairwise.prop.test).* The risks of each hippoboscid species to be infected with only one or with two concurrent pathogens in the same individual (positive/negative) were evaluated with logistic regression models (*glm,* family: “binomial”) using louse fly species as explanatory variable to calculate odds ratios (OR) and 95% confidence intervals (95% CI). A level of *P* < 0.05 was considered significant.

## Results

### Species and DNA barcoding of collected hippoboscid flies

**Fig. 2:**
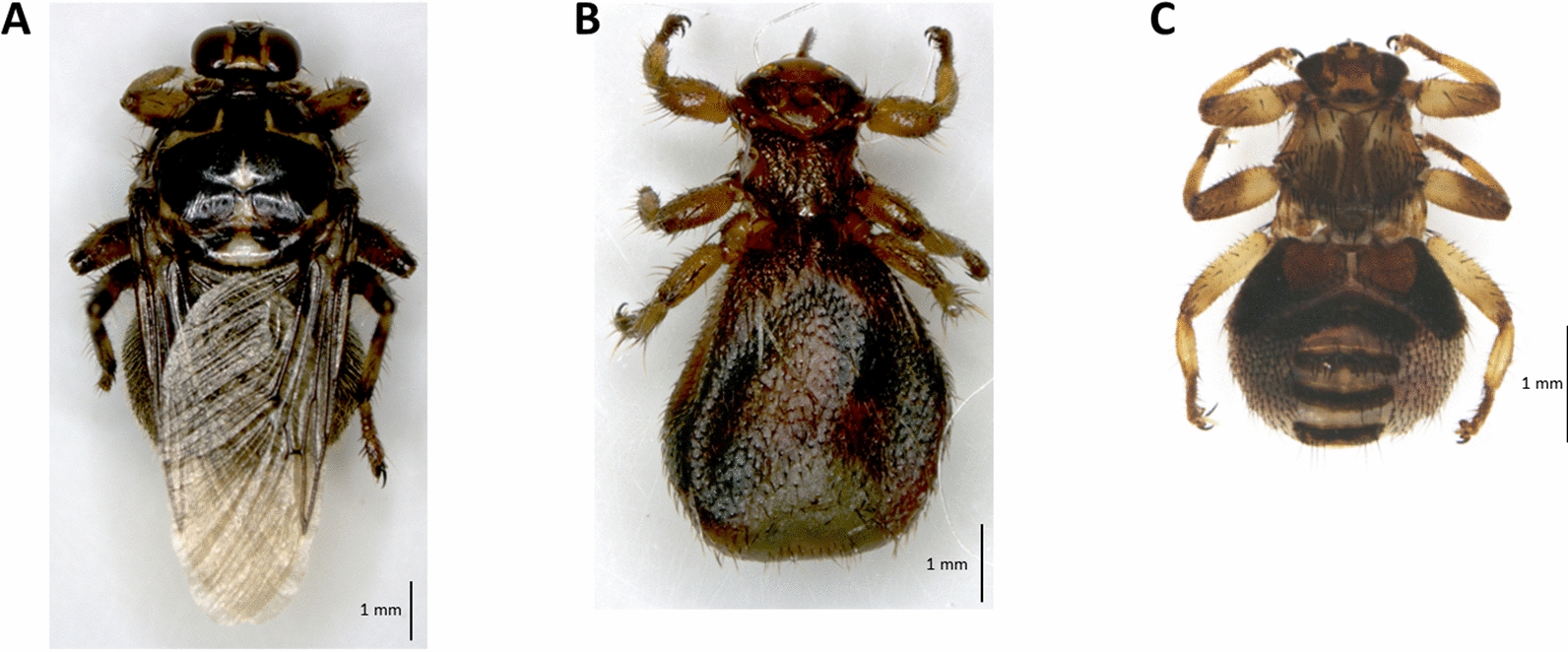
Hippoboscid flies for molecular pathogen screening. Representative individuals of *Hippobosca equina* (**A**), *Melophagus ovinus* (**B**) and *Lipoptena cervi* (**C**) collected from domestic and wild ruminants in Austria

A total of 282 louse flies were collected from naturally infested cattle (*n* = 25), sheep (*n* = 3) and red deer (*n* = 12) in different regions of Austria. The hippoboscids were identified as *Hippobosca equina* (*n* = 62; Fig. 2A) collected from cattle, *Melophagus ovinus* (*n* = 100; Fig. 2B) from sheep and *L. cervi* (*n* = 120; Fig. 2C) from red deer. Barcode analyses in BOLD of 21 individual hippoboscids (*H. equina*, *n* = 5; *M. ovinus*, *n* = 5; *L. cervi*, *n* = 11) revealed that the *COI* sequences of each louse fly species clustered within a respective Barcode Index Number with sequences previously reported from Europe, Northern Africa and Asia for *H. equina* (BOLD:AAX0882), *M. ovinus* (BOLD:AAX4771) and *L. cervi* (BOLD:ABX1452). Obtained *COI* sequences from the barcoded hippoboscids were submitted to GenBank under the following accession numbers: ON129173, ON129175, ON129176, ON129178, ON129181 (*H. equina*); ON129174, ON129177, ON129179, ON129180, ON129182 (*M. ovinus*); ON341137 – ON341147 (*L. cervi*).

### Molecular pathogen screening

**Fig. 3:**
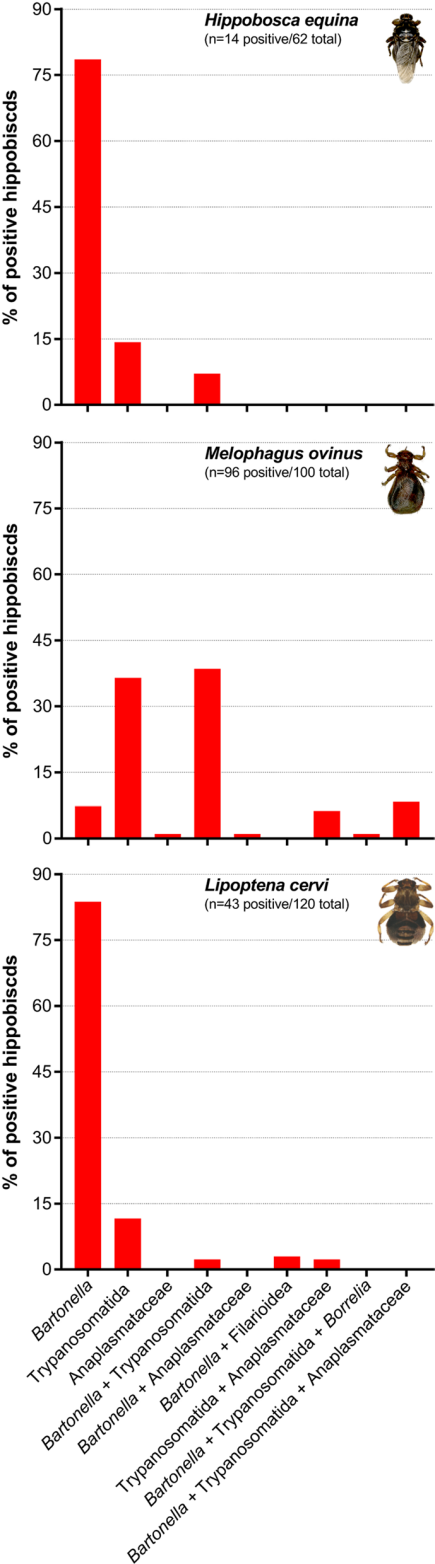
Single and co-infections in pathogen-positive hippoboscid flies. Percentage of hippoboscid flies positive to one, two or three pathogens in the same individual of *Hippobosca equina*, *Melophagus ovinus* and *Lipoptena cervi* collected from domestic and wild ruminants in Austria

Molecular screening revealed pathogen DNA in 153/282 (54.3%) of the collected hippoboscids, with substantial differences between louse fly species (Table 2). The sheep ked *M. ovinus* were significantly more frequently infected with pathogens in comparison with *L. cervi* (*χ*^2^ = 73.944, *df* = 1, *P* < 0.001) and *H. equina* (*χ*^2^ = 82.315, *df* = 1, *P* < 0.001), whereas no difference in total infection rate was observed between *L. cervi* and *H. equina* (*χ*^2^ = 2.7502, *df* = 1, *P* = 0.09; Table 2). Of all 153 positive individuals among the three hippoboscid species, 97 carried only one pathogen (63.4%), 47 were infected with two different pathogens (30.7%), and three distinct pathogens were confirmed in nine *M. ovinus* specimens (5.9%). The percentages of hippoboscids from each species infected with single or multiple pathogens in the same individuals are illustrated in Fig. 3. From the three hippoboscid species, *M. ovinus* had significantly higher odds to be infected with at least one pathogen compared with *H. equina* (OR *M. ovinus*: 2.8 [1.4–5.9], *P* < 0.01), whereas *L. cervi* had slightly higher odds to be infected with a single pathogen compared with *H. equina* (OR *L. cervi* [95% CI] 1.9 [0.95–4.05], *P* = 0.07). *Melophagus ovinus* was significantly most likely to be infected with two concurrent pathogens compared with *H. equina* (OR = 47.1 [9.8–849.2], *P* < 0.001). No differences were observed in the risk of carrying two pathogens between *L. cervi* and *H. equina* (OR = 1.02 [0.1–22.1, *P* > 0.5].

**Table 2:** Pathogens detected by molecular screening in hippoboscid flies (Diptera: Hippoboscidae) collected from domestic and wild ruminants in Austria

Host (*n*)	Hippoboscid flies (*n*)	Hippoboscids positive to pathogens/total hippoboscids screened per species (% positive)						
		Total infection rate (Positive to ≥ 1 pathogen[s])	*Bartonella*	Trypanosomatida	Anaplasmataceae	*Borrelia*	Filarioidea	Piroplasmida
Cattle (*n* = 25)	*Hippobosca equina* (*n* = 62)	14/62 (22.5%)^a^	12/62 (19.3%)^a^	3/62 (4.8%)^a^	0/62	0/62	0/62	0/62
Sheep (*n* = 3)	*Melophagus ovinus* (*n* = 100)	96/100 (96%)^b^	54/100 (54%)^b^	87/100 (87%)^b^	16/100 (16%)	1/100 (100%)	0/100	0/100
Red deer (*n* = 12)	*Lipoptena cervi* (*n* = 120)	43/120 (35.8%)^a^	38/120 (31.6%)^a^	6/120 (5%)^a^	0/120	0/120	1/120 (0.8%)	0/120

^a,b^Different letters within the same column (pathogen) represent statistically significant differences in the proportion of pathogen-positive individuals between hippoboscid species (*P* < 0.05)

*Bartonella* spp. was detected in 36.9% (104/282) of the investigated hippoboscids, followed by Trypanosomatida in 34.0% of all louse flies (96/282). Individuals of *M. ovinus* were found more frequently infected with *Bartonella* spp. in comparison with *H. equina* (*χ*^2^ = 17.619, *df* = 1, *P* < 0.001) and with *L. cervi* (*χ*^2^ = 10.283, *df* = 1, *P* < 0.001). The sheep ked *M. ovinus* were also more often infected with Trypanosomatida compared to *H. equina* (χ^2^ = 101.33, df = 1, *P* < 0.001) and to *L. cervi* (*χ*^2^ = 146.95, df = 1, *P* < 0.001). Only *M. ovinus* individuals were positive to Anaplasmataceae in 5.7% (16/282; 5.7% of all investigated hippoboscids) and less than 1% of the louse flies were positive for *Borrelia* spp. (one *M. ovinus* specimen) and Filarioidea (one *L. cervi* specimen). All investigated hippoboscids were negative for Piroplasmida. The sequences of the pathogens screened in the present study were deposited in GenBank under the following acc. no.: ON668330 (*Borrelia* spp.), ON678056 (Filarioidea), ON637624 – ON637640 (Trypanosomatida) and OP198738 – OP198806 (*Bartonella* spp.).

**Fig. 4:**
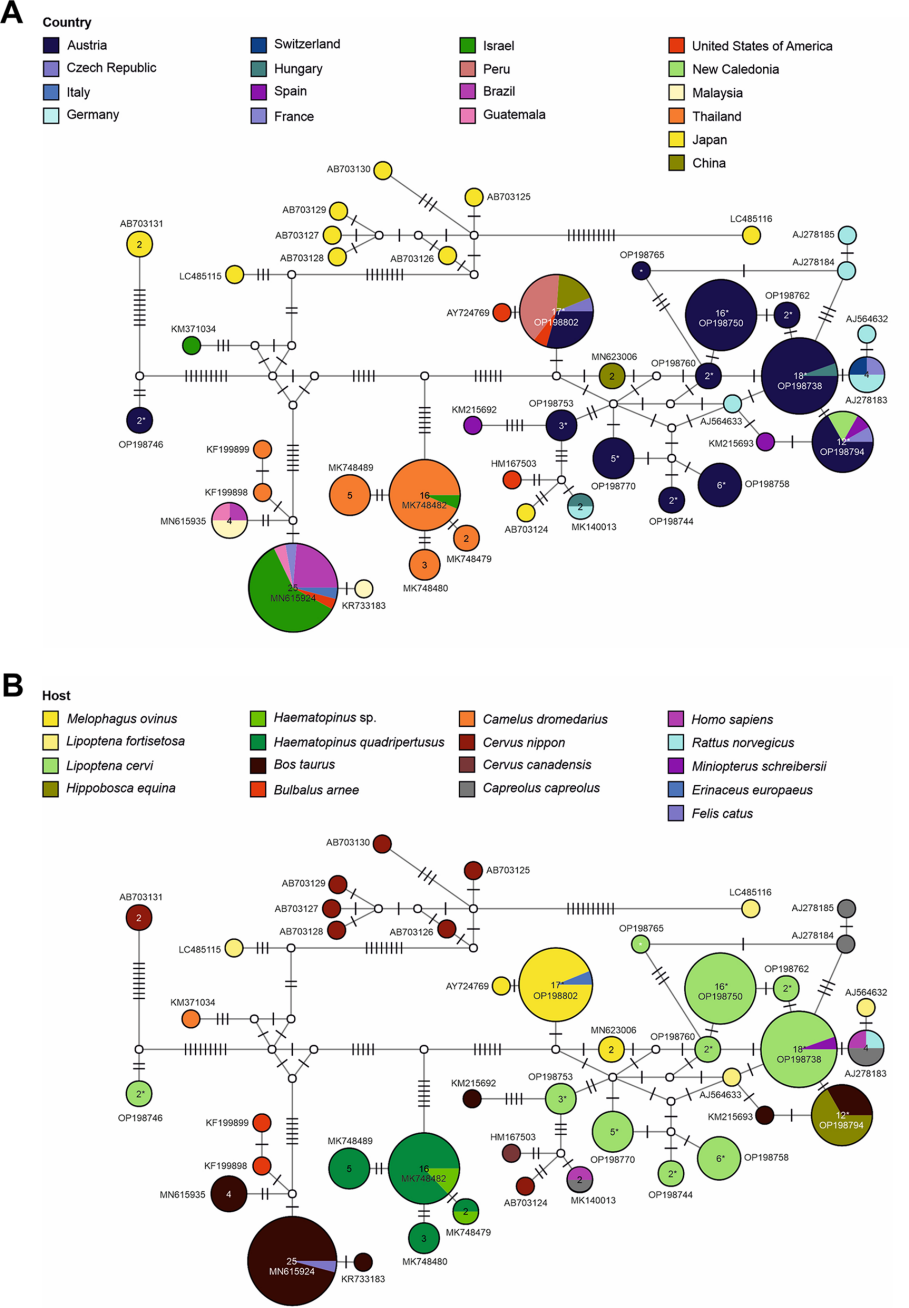
Genetic diversity of *Bartonella* detected in hippoboscid flies. Median-joining haplotype network of the *gltA* sequences (338 bp) of selected *Bartonella* spp. from the present and previous studies showing their geographical distribution (**A**) and the reported hosts (**B**). Circles represent haplotypes, and numbers within the circles represent the number of individuals. If no number is shown, then only one individual is represented. Labels next to circles specify representative GenBank accession numbers of the haplotypes; white circles represent intermediate nodes; bars on branches interconnecting haplotypes represent the number of substitutions. Asterisks mark haplotypes detected in the present study

*Bartonella* spp. were the most common infectious agents detected in *H. equina* and *L. cervi*, and the second most frequently identified pathogen in *M. ovinus* after trypanosomatids (Table 2, Fig. 3). In *L. cervi*, several isolated *Bartonella* spp. *gltA* (citrate synthase gene) sequences showed 100% identity with a *Bartonella* sp. strain isolated from the bat *Miniopterus schreibersii* in Hungary (MK140014; see Additional file 2: Table S1). Furthermore, various *Bartonella* spp. *gltA* sequences from *L. cervi* showed > 99% identity with reported sequences of *B. schoenbuchensis* isolated from roe deer (*Capreolus capreolus*; GenBank acc. no.: AJ278184; AJ278185) and from *L. cervi* (AJ564634; AJ564635; Additional file 2) in Germany. Haplotype network analyses of the isolated *Bartonella* spp. *gltA* sequences from *L. cervi* revealed ten novel strains not previously reported (Fig. 4 and Additional file 1), including one strain (OP198738) identical to a *Bartonella* sp. sequence isolated from the bat *M. schreibersii* in Hungary (Fig. 4). In contrast to the broad diversity of *Bartonella* spp. strains detected in *L. cervi*, only one haplotype was identified in sequences isolated from *H. equina* (OP198794), which was 100% identical to sequences of *Bartonella chomelii* reported from Spain, France and New Caledonia (KM215691; KM215690; JN646657; Fig. 4 and Additional file 1). The *Bartonella* spp. sequences identified in *M. ovinus* (OP198802) showed 100% identity to sequences of *Candidatus* *Bartonella melophagi* from *M. ovinus* in Peru, the USA and China and from a European hedgehog (*Erinaceus europaeus*) in Czechia (MZ089835; MT154632; Fig. 4; Additional file 2). In the BI tree (Additional file 1), the sequences of *B. chomelii*, *Candidatus* *B. melophagi* and *Bartonella* sp. clustered in one clade with other *Bartonella* spp. previously reported from ruminants (BI posterior probability [BI pp] = 1.0, ML bootstrap value [ML bs] = 99). Most sequences of *B. chomelii*, *Candidatus* *B. melophagi* and *Bartonella* spp. detected in the present study clustered in one subclade with *B. chomelii*, *B. schoenbuchensis*, *B. capreoli* and *Bartonella* spp. sequences (BI = 1, ML = 100; Additional file 1). Only one *Bartonella* sp. sequence from *L. cervi* (OP198746) was placed in a separate sister clade together with *B. bovis* and *Bartonella* spp. (BI = 0.98, ML = 85).

**Fig. 5:**
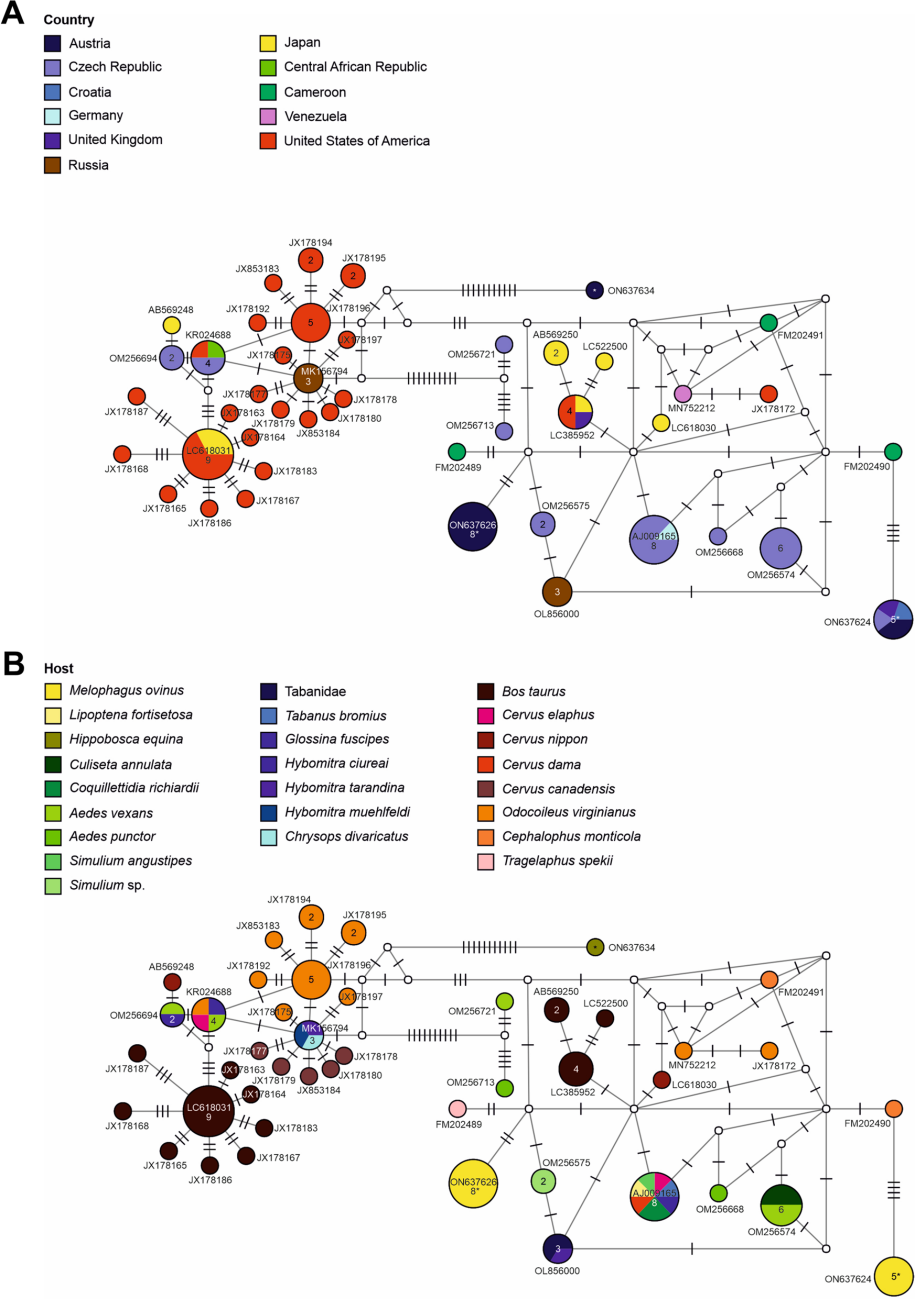
Genetic diversity of *Trypanosoma* detected in hippoboscid flies. Median-joining haplotype network of the *18S* rRNA sequences (779 bp) of selected *Trypanosoma* spp. from the present and previous studies showing their geographical distribution (**A**) and the reported hosts (**B**). Circles represent haplotypes and numbers within the circles represent the number of individuals. If no number is shown, then only one individual is represented. Labels next to circles specify representative GenBank accession numbers of the haplotypes; white circles represent intermediate nodes; bars on branches interconnecting haplotypes represent the number of substitutions. Asterisks mark haplotypes detected in the present study

Trypanosomatid sequences (*18S* rRNA) were detected in all hippoboscid species and represented the most common pathogens in *M. ovinus* with 87% positive individuals (Table 2, Fig. 3). *Trypanosoma* spp. sequences isolated from *M. ovinus* were 100% identical to *18S* sequences of *Trypanosoma melophagium* from Czechia, Croatia and the UK (OM256700; HQ664912; FN666409). The DNA haplotype network analysis revealed two distinct strains of *T. melophagium*: a new strain (ON637626) and a second one (ON637624) identical to *T. melophagium* sequences isolated from *M. ovinus* in Croatia, UK and Czechia (Fig. 5 and Additional file 1). Only three *H. equina* individuals (two from Saalfelden and one from Eisenstadt) were positive for trypanosomatids, featuring a *Trypanosoma* sp. (ON637634) that clustered together with other trypanosomatids of ruminants, including *Trypanosoma theileri*, *T. trinaperronei*, *T. melophagium*, *T. cervi* and *Trypanosoma* spp. (Fig. 5 and Additional file 1). The *Trypanosoma* sequences obtained from *H. equina* shared  > 98% similarity with those of *Trypanosoma* cf. *cervi* isolated from white-tailed deer in the USA (JX178196), *Trypanosoma* sp. from horse flies in Russia (MK156792-MK15794) and *T. theileri* obtained from tsetse flies in the Central African Republic (KR024688). While the trypanosomatid sequences isolated from *L. cervi* showed  > 99% identity to sequences of non-parasitic kinetoplastids of the genus *Bodo* from the UK and USA (AY425015; AY028450).

Anaplasmataceae sequences (*16S* rRNA) were only detected in 16 M*. ovinus* individuals, featuring sequences identical to those of several *Wolbachia* strains, including a strain previously isolated from *M. ovinus* (MF461472; KY224164; KY224163). *Borrelia* spp. (*16S* rRNA) was detected in a single *M. ovinus* and had a 93.7% similarity with a reported *Borrelia* sp. (CP043682) isolated from ticks associated with passeriform birds. Finally, one *L. cervi* individual featured the *COI* sequence of an unknown onchocercid nematode (Filarioidea), most similar (95.1%) to *Mansonella perforata* isolated from Sika deer (*Cervus nippon*) in Japan (AM749265).

## Discussion

Here, we confirmed the molecular presence of various pathogens in blood-sucking hippoboscid flies infesting domestic and wild ruminants in Austria. The three louse fly species collected and investigated, *L. cervi*, *M. ovinus* and *H. equina*, have a widespread distribution in Europe [1, 50]. In the present study, *L. cervi* and *M. ovinus* were collected from their primary hosts, deer and sheep, respectively, whereas all *H. equina* were obtained from cattle, one of their facultative hosts [17]. The three investigated hippoboscid species differed in their total infection rates and infection prevalences to the different pathogen-groups, with *M. ovinus* specimens being significantly more infected than *L. cervi* and *H. equina* individuals to at least one pathogen (regardless of pathogen group), to *Bartonella* spp. and to trypanosomatids. Sheep keds also had a higher risk of being infected with two concurrent pathogen groups compared with *L. cervi* and *H. equina*. However, detailed molecular analyses revealed different pathogens (within each pathogen group) infecting each louse fly species; therefore, a comparison of the prevalences of the same pathogen between hippoboscids and their animal hosts is not possible. The different pathogens identified in the three hippoboscid species, and the probable role of these hippoboscids as vectors of the identified pathogens, are discussed below.

*Bartonella* spp. were the most frequently detected pathogens in *H. equina* and *L. cervi*, and the second most in *M. ovinus.* All *Bartonella* spp. in our study corresponded phylogenetically to species of the *Bartonella* linage II associated with strains that infect domestic and wild ruminants [51]. *Bartonella* spp. were first described in *H. equina*, *L. cervi* and *M. ovinus* almost 20 years ago [20, 21], with growing evidence pointing at the role of these hippoboscids as *Bartonella* vectors [28, 30, 52–57]. Importantly, we found ten distinct and previously unreported *Bartonella* spp. strains in *L. cervi* collected from red deer. Seven of these *Bartonella* spp. strains were highly similar (> 99%) to *B. schoenbuchensis*, a widespread pathogen infecting the midgut of deer keds [20, 28, 54]. *Bartonella schoenbuchensis* has been molecularly detected in blood and tissues samples from various wild ungulates, including red deer, roe deer and moose (*Alces alces*), all natural hosts for *L. cervi* [1, 28, 58–61]*.* Our results suggest that *B. schoenbuchensis* and related *Bartonella* spp. strains are common in *L. cervi* in Austria and may also be circulating in the local wild red deer populations. This is noteworthy in a One-Health context, considering that *B. schoenbuchensis* can be transmitted to humans, as described by a report of bacteremia in a patient suffering from fatigue, muscle pain and fever following a tick bite [62]. Moreover, *B. schoenbuchensis* has been suggested as the etiological agent of deer ked dermatitis in humans bitten by *L. cervi* [20], with similar clinical signs to cat scratch disease caused by the zoonotic *Bartonella henselae* [54, 63]. Therefore, the presence and distribution of *B. schoenbuchensis* in wild deer, deer keds and potentially other arthropod vectors in Austria warrant confirmation. Additionally, one *Bartonella* sp. strain isolated from deer keds in our study matched with a previously reported *Bartonella* sp. sequence detected in the common bent-wing bat *M. schreibersii* [64]. This *Bartonella* sp. and the *B. schoenbuchensis*-like strains identified in our study clustered together with *B. schoenbuchensis* and *B. chomelii* in the DNA haplotype network analysis. The other two *Bartonella* spp. strains detected in *L. cervi* clustered in a separate subclade and were highly similar to sequences of *Candidatus* *B. melophagi* reported from *M. ovinus* [65] and to *Bartonella* sp. isolated from Sika deer [66]. The diversity of *Bartonella* spp. lineages detected in deer keds in the present study and the presence of co-infections with two different *Bartonella* spp. lineages in several individuals indicate that *L. cervi* are reservoirs for a wide range of *Bartonella* spp. strains in Austria. Recent studies have also reported the recovery of several *Bartonella* spp. strains with zoonotic potential in deer keds (*Lipoptena cervi* and *L. fortisetosa*) and in cervids across Europe [27, 31, 33, 67], implying that these wild ungulates may act as reservoir hosts for these pathogens. Consequently, considering the common occurrence of wild cervids in Austria and the increasing reports of deer keds attacking humans in Europe [3, 6, 13, 68], it is imperative to further expand the monitoring and identification of zoonotic *Bartonella* spp. in deer keds and cervid populations.

*Bartonella chomelii* was the sole *Bartonella* species detected in *H. equina* collected from cattle in the present study. *Bartonella chomelii* was first described as a distinct *Bartonella* species from blood samples of cows in France [69], and subsequent reports in different countries confirmed its presence in both cattle [57, 70, 71] and *H. equina* [21, 56, 57]. Recently, molecular screenings also identified *B. chomelii* in ticks collected from rodents and dogs [72, 73]. In contrast, *B. chomelii* has not been detected in horses or other equids (the primary hosts of *H. equina*) or in *H. equina* parasitizing horses [21, 71]. It has been suggested that cattle could be accidental hosts for *B. chomelii*, which may be more closely related to *Bartonella* spp. from wild ruminants than strains isolated from domestic cattle such as *B. bovis* [69]. Considering that this is the first report of *B. chomelii* in Austria, further studies are needed to understand the occurrence and potential impact of this pathogen in cattle and wild ruminant populations. Previous work has suggested a higher prevalence of *B. chomelii* in older cattle (> 2 years old) and livestock managed in mountain pastures (> 600 m above sea level) [57, 71]. In the present study, *B. chomelii*-positive *H. equina* were collected from cattle grazing on mountain grasslands (~ 1000 to 1450 m above sea level; data not shown), located in the Hohe Tauern Alps of Salzburg [34], which suggests that animals during alpine grazing may be at risk of infections with *B. chomelii*, although this remains to be confirmed. To date, *B. chomelii* has not been demonstrated to induce disease in cattle, but infections with the related species *B. bovis* have been associated with bovine endocarditis [74].

Our results confirmed that the *Bartonella* spp. sequences detected in *M. ovinus* belonged to *Candidatus* B. melophagi, one of the most common pathogens in sheep ked populations [21, 53, 56]. Once thought to be only an endosymbiont of sheep keds not transmissible to ruminants [21], new evidence has confirmed the presence of *Candidatus* B. melophagi in sheep, including its successful culture from ovine blood, thus suggesting that sheep can serve as a host reservoir for this pathogen, with *M. ovinus* as its likely vector [55, 75]. However, it is still unclear whether *Candidatus* B. melophagi can cause clinical disease in sheep and whether *M. ovinus* is a competent vector transmitting these bacteria. Importantly, *Candidatus* B. melophagi was isolated from blood samples of two human patients presenting non-specific symptoms such as cardiovascular problems, pain and fatigue [76]. Despite these two patients having declared frequent contact with domestic and wild animals, there was no evidence of a possible route of infection or an actual causality between *Candidatus* B. melophagi infections and the clinical symptoms [76]. Therefore, the zoonotic potential of *Candidatus* B. melophagi and the role of *M. ovinus* as its vector require further elucidation.

In the present study, *T. melophagium* was the sole trypanosomatid species detected in *M. ovinus*, with high infection rate among the investigated sheep keds. *Trypanosoma melophagium* has been known to infect sheep keds and sheep for over a century [18, 77], and the present study adds to the few previous molecular genetic surveys confirming its high prevalence in *M. ovinus* collected from sheep in Scotland [22], Croatia [25] and recently Czechia [78]. Phylogenetic studies have concluded that *T. melophagium* is a single species restricted to *M. ovinus* and sheep; it is a member of the subgenus *Megatrypanum*, which includes other host-restricted pathogens infecting domestic and wild ruminants such as *Trypanosoma theileri* in cattle [25, 79]. Early works reported absent or very low parasitemia of *T. melophagium* in sheep infested by *M. ovinus* carrying this pathogen, and it has been suggested that sheep may become infected with *T. melophagium* merely by oral ingestion of sheep keds [18, 25]. To date, there is no evidence that *T. melophagium* can cause disease in sheep or be transmitted to other ruminant or mammalian species, and it has been proposed that it is non-pathogenic for infected *M. ovinus* [80]. Nevertheless, our findings suggest that sheep infested with *M. ovinus* in Austria could be infected with *T. melophagium*, and this should be confirmed, particularly in sheep farms with low or no use of ectoparasiticides for the control of sheep keds (e.g. organic farms), as previously described [25].

Our work identified a *Trypanosoma* sp. strain in three *H. equina* individuals collected from cattle in two different states of Austria (Salzburg and Burgenland). To the best of the authors’ knowledge, this is the first report of *Trypanosoma* sp. in *H. equina*, thus pointing at a potential role of this hippoboscid as a novel vector of animal trypanosomatids. In our phylogenetic analysis, this *Trypanosoma* sp. strain clustered together with sequences of the *T. theileri* group and was highly similar to *Trypanosoma* cf. *cervi* sequences isolated from white-tailed deer (*Odocoileus virginianus*) in the USA [81], with *T. theileri*-like strains from the horse flies *Hybomitra tarandina*, *Chrysops divaricatus* and *Hybomitra muehlfeldi* in Russia [82] and with *T. theileri* obtained from the tsetse fly *Glossina fuscipes* in the Central African Republic [83]. A previous study in Austria revealed a high prevalence of species belonging to the *T. theileri*/*cervi* complex in mosquitoes, suggesting a widespread distribution of these pathogens in animal hosts, potentially wild ruminants [84]. Therefore, the *Trypanosoma* sp. strain detected in *H. equina* in the present work might belong to the *T. theileri* group/complex, which may be elucidated by future molecular studies monitoring trypanosomatids in *H. equina* and cattle using various target genes. Furthermore, considering that *T. theileri* has not yet been reported to infect cattle in Austria but is present in neighboring countries [85, 86], the confirmation of *T. theileri* in vectors such as *H. equina* and tabanid flies, as well as in Austrian cattle, is warranted. Molecular genetic studies on trypanosomatids such as *T. theileri*-like strains have also been reported in the deer keds *Lipoptena fortisetosa* and *L. cervi* in Poland and Czechia [29, 78], but they were not detected in the current study. Regarding trypanosomatids detected in the deer ked *L. cervi*, we identified sequences highly similar to *Bodo* sp., which are non-parasitic kinetoplastids (suborder Bodonina) present in soil and water. Although *Bodo* spp. were isolated from bat ectoparasites and from the woylie *Bettongia penicillate*, an Australian marsupial, these findings were associated with environmental contamination of the mammalian hosts rather than infection [87, 88]. Therefore, we cannot exclude environmental contamination in our samples and further studies are needed to evaluate the role of *Bodo* sp. in hippoboscids.

In the present study, Anaplasmataceae were only detected in sheep keds and identified as *Wolbachia* spp. strains, which are known endosymbionts of nematodes and arthropods, including hippoboscids such as *H. equina* and *M. ovinus* [56, 65, 89, 90]. The *16S* sequences of *Wolbachia* were identical to those of strains previously isolated from *M. ovinus* [91]. To date, the specific role or effects of *Wolbachia* on *M. ovinus* and other hippoboscid flies are unknown [90]. No pathogenic Anaplasmataceae were detected in the investigated hippoboscids, although *M. ovinus* and *L. cervi* can be infected with *Anaplasma ovis* [23, 92] and *A. phagocytophilum* [24], respectively. Considering that the zoonotic *A. phagocytophilum* has been confirmed to infect wild cervids and bovids in Austria [33, 93, 94], further work is needed to expand the monitoring of pathogenic Anaplasmataceae in hippoboscid flies and their ruminant hosts, whereas the single *Borrelia*-positive hippoboscid detected was a *M. ovinus* individual infected with a previously unreported strain 93.7% similar to a *Borrelia* isolate denominated A-FGy-1 and to *Candidatus* Borrelia mahuryensis isolated from neotropical passerine-associated ticks [95]*.* An early work confirmed the presence of *Borrelia burgdorferi* sensu lato, one of the causative agents of Lyme disease in humans, in *M. ovinus* [96]*.* The *Borrelia* sp. *16S* sequence obtained in our study was 92.9% similar to two *B. burgdorferi* sequences uploaded to GenBank (AJ224138 and AJ224134). Considering the potential zoonotic risk of *B. burgdorferi*, new studies should characterize the identity and distribution of *Borrelia* spp. in sheep keds in Austria. Finally, only one *L. cervi* individual was positive for Filarioidea, featuring the *COI* sequence of an unknown onchocercid with a genetic similarity of 95% to the dermal filaroid *Mansonella perforata*, previously isolated from Sika deer [97, 98]. Whether this onchocercid sequence belongs to *M. perforata* or to another, not previously sequenced *Mansonella* species needs to be confirmed, as well as their potential distribution in red deer and deer ked populations in Austria.

Since the hippoboscid flies collected in the present study were isolated following a convenience sampling in selected farms with expected high infestation prevalence (sheep/cattle) and hunted animals as part of a tuberculosis surveillance program (deer), our results do not allow an accurate estimation of the country-wide prevalence of hippoboscid-associated pathogens in Austria. However, our data describe the widespread presence of pathogens in louse flies infesting ruminants in various geographic regions. Therefore, our findings support the need for the monitoring of hippoboscids and hippoboscid-borne diseases infecting domestic and wild ruminants in Austria, and potentially in other European regions, on a wider scale. Other pathogens previously reported in hippoboscid flies but not surveyed in the present study could also be included in future molecular surveys such as *Rickettsia* spp., *Theileria ovis*, *Acinetobacter* spp., *Bacillus* spp., *Staphylococcus* spp., Blue-tongue virus, Border Disease virus and *Corynebacterium pseudotuberculosis* [17]. Furthermore, the impact of climate change on the distribution and seasonal dynamics of louse flies should be investigated. Clearly, monitoring of potential zoonotic pathogens in (wild) animal reservoirs needs to be expanded in Austria, as indicated by the recent confirmation of *Anaplasma phagocytophilum* and *Babesia* spp. in wild ungulates [33].

This work contributes to the ongoing research efforts towards clarifying the role of blood-sucking hippoboscid flies as vectors of infectious agents of veterinary and medical importance. However, the presence of hippoboscid-associated pathogens confirmed by PCR and sequencing does not prove the vector competence of the investigated louse fly species for these pathogens, and only suggests their vector potential. Vector competence is the ability of arthropods to acquire and transmit the infective stage of a pathogen to a vertebrate host, including the pathogen’s replication within the vector. It requires conclusive experimental, epidemiological and clinical evidence of the pathogen’s transmission from the vector to the host, and the subsequent infection [99, 100]. For hippoboscids, there is evidence that *Bartonella* spp. can be vertically transmitted in *L. cervi*, with the pathogen being detected in adult deer keds blood feeding on ruminants, in pupae and in unfed adult flies that had not yet started to feed on blood [54]. However, whether *L. cervi* can transmit *Bartonella* spp. to a vertebrate host remains to be confirmed. Moreover, it should still be clarified whether the pathogens detected in *L. cervi* (e.g. *Bartonella* spp.) have zoonotic potential, how widespread these infectious agents are in wild ruminant populations in Austria and whether these animals may act as reservoirs for pathogens that could be vectored by deer keds. This is critically important considering the potential exposure of humans to bites by *Bartonella*-infected *L. cervi* during working or leisure activities (e.g. hunters, forestry workers, hikers).

## Conclusions

In conclusion, molecular genetic screening confirmed the presence of several pathogens in three hippoboscid species infesting domestic and wild animals in Austria, with some potentially representing emerging zoonotic risks such as *Bartonella* spp. We report several novel pathogen sequences in hippoboscids that can contribute to the ongoing research efforts to understand the vector role of louse flies, including the first detection of *Trypanosoma* spp. in *H. equina*. Expanded monitoring of hippoboscids and hippoboscid-borne pathogens is warranted to clarify the distribution and impact of these ectoparasites as vectors of emerging infectious agents of public and animal health importance in a One-Health context.

## Supplementary Information

**Additional file 1: Figure S1.** Bayesian inference tree featuring gltA sequencesof selected *Bartonella* spp. Nodes are marked with Bayesian inference posterior probabilities and maximum likelihood bootstrap values. Clades which are marked with a red bar were used for calculation of the median-joining haplotype networks containing the sequences obtained in this study. Scale bar indicates the expected mean number of substitutions per site according to the model of sequence evolution applied. **Figure S2**. Bayesian inference tree featuring 18S rRNA sequencesof selected *Trypanosoma* spp. Nodes are marked with Bayesian inference posterior probabilities and maximum likelihood bootstrap values. Clades which are marked with a red bar were used for calculation of the median-joining haplotype networks containing the sequences obtained in this study. Scale bar indicates the expected mean number of substitutions per site according to the model of sequence evolution applied.

**Additional file 2: Table S1.** Blast analysis of Bartonella spp. sequencesobtained from kedscollected from domestic and wild ruminants in Austria.

## Data Availability

All data generated or analysed during this study are included in this published article [and its supplementary information files].
