# Supplementary material for: Molecular pathogen screening of louse flies (Diptera: Hippoboscidae) from domestic and wild ruminants in Austria
Source: Parasit Vectors. 2023 Jun 2;16:179. doi: 10.1186/s13071-023-05810-4 (PMC10236838; doi:10.1186/s13071-023-05810-4)
Supplement: Supplementary file 1 — Additional file 1: Figure S1. Bayesian inference tree featuring gltA sequencesof selected Bartonella spp. Nodes are marked with Bayesian inference posterior probabilities and maximum likelihood bootstrap values. Clades which are marked with a red bar were used for calculation of the median-joining haplotype networks containing the sequences obtained in this study. Scale bar indicates the expected mean number of substitutions per site according to the model of sequence evolution applied. Figure S2. Bayesian inference tree featuring 18S rRNA sequencesof selected Trypanosoma spp. Nodes are marked with Bayesian inference posterior probabilities and maximum likelihood bootstrap values. Clades which are marked with a red bar were used for calculation of the median-joining haplotype networks containing the sequences obtained in this study. Scale bar indicates the expected mean number of substitutions per site according to the model of sequence evolution applied. [file 13071_2023_5810_MOESM1_ESM.pdf]

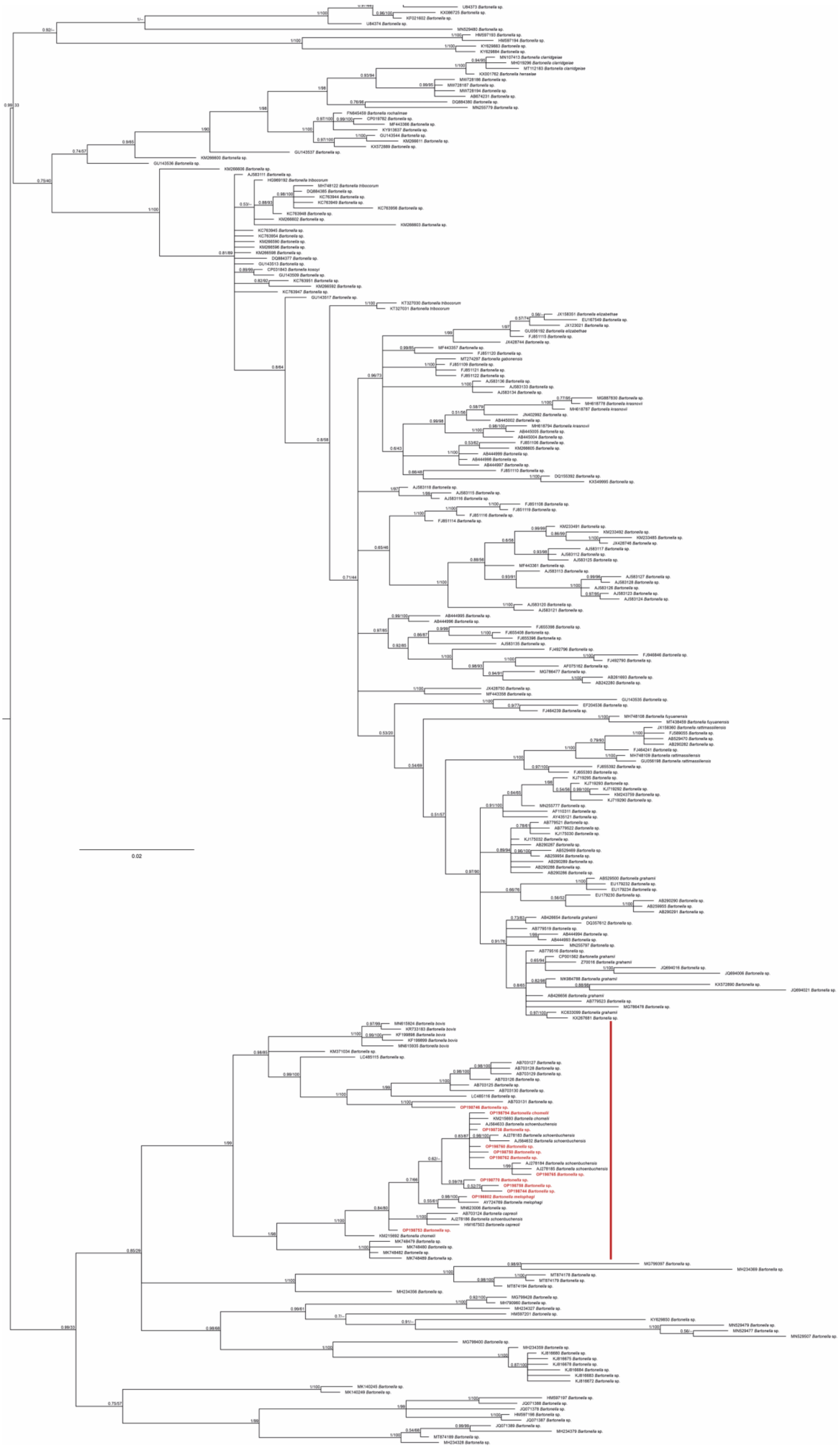

Supplementary Fig. 1. Bayesian inference tree featuring gltA sequences (338 bp) of selected *Bartonella* spp. Nodes are marked with Bayesian inference posterior probabilities and Maximum likelihood bootstrap values. Clades which are marked with a red bar were used for calculation of the median-joining haplotype networks containing the sequences obtained in this study (font colour in red). Scale bar indicates the expected mean number of substitutions per site according to the model of sequence evolution applied

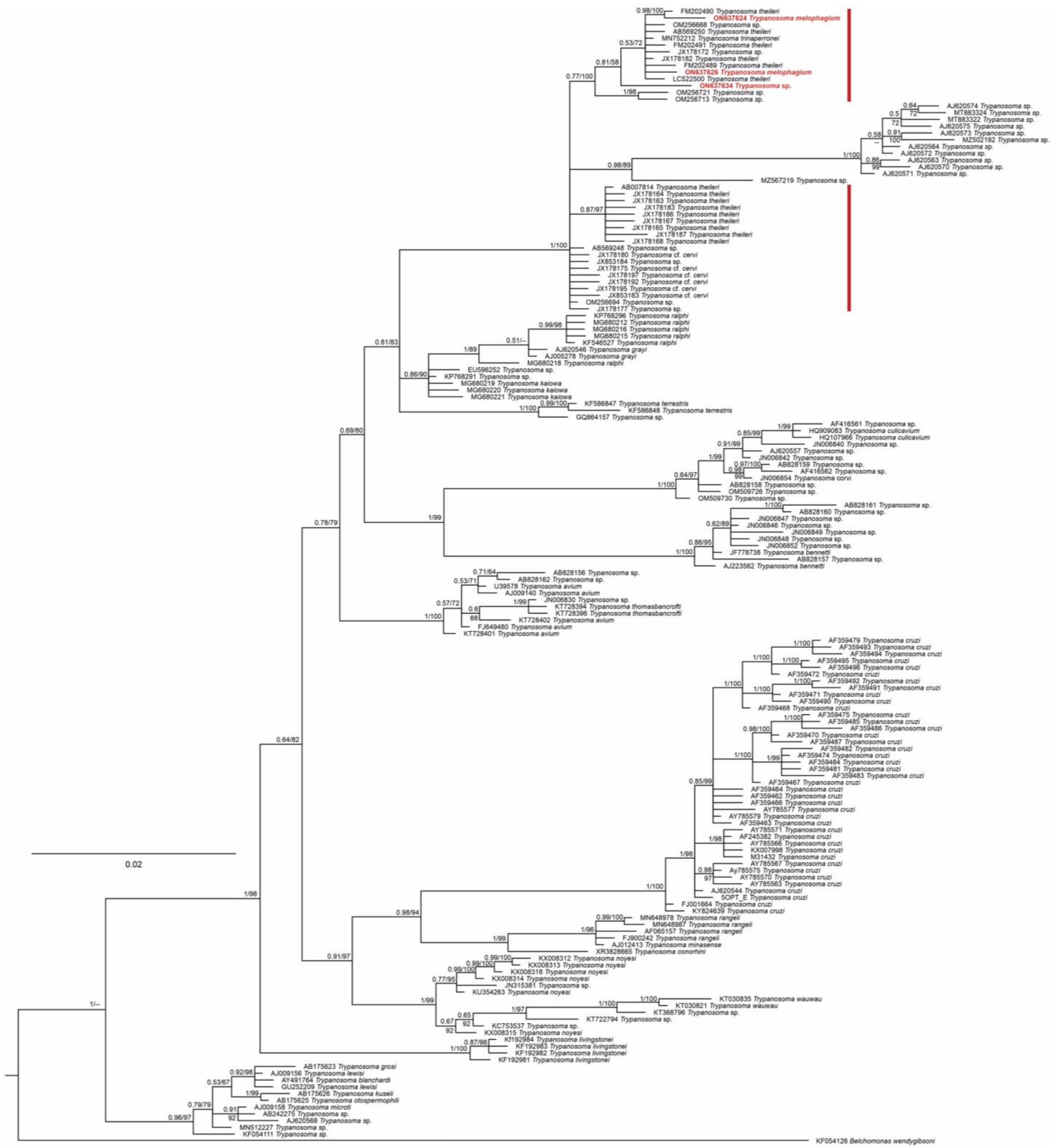

Supplementary Fig. 2. Bayesian inference tree featuring 18S rRNA sequences (701 bp) of selected *Trypanosoma* spp. Nodes are marked with Bayesian inference posterior probabilities and Maximum likelihood bootstrap values. Clades which are marked with a red bar were used for calculation of the median-joining haplotype networks containing the sequences obtained in this study (font colour in red). Scale bar indicates the expected mean number of substitutions per site according to the model of sequence evolution applied.
